# *OPCML is* hypermethylated in a subset of patients with metaplastic changes in their esophagus

**DOI:** 10.1186/s40364-018-0150-y

**Published:** 2018-12-07

**Authors:** Natalia Castaño-Rodríguez, Georgia L. Popple, Gloria Liliana Porras-Hurtado, José Luis Cardona-Deazza, Juan José Montoya-Martinez, Antonio Javier Cadavid-Velez, Héctor William Toro-Hidalgo, Alba Ruth Cobo-Alvarado, Ofelia del Socorro Hincapié-Rincón, Stephen M. Riordan, Nadeem O. Kaakoush

**Affiliations:** 1 School of Biotechnology and Biomolecular Sciences, UNSW Sydney, Sydney, NSW 2052 Australia; 2 Clínica Comfamiliar Risaralda, Pereira, Risaralda Colombia; 3 Gastrointestinal and Liver Unit, The Prince of Wales Hospital, Randwick, NSW 2031 Australia; 4 School of Medical Sciences, Faculty of Medicine, UNSW Sydney, Kensington, NSW 2052 Australia

**Keywords:** *OPCML*, CpG methylation, Dysplasia, Adenocarcinoma

## Abstract

*OPCML* hypermethylation is considered a promising cancer biomarker. We examined methylation levels in the first exon of *OPCML* in two patient cohorts within the esophageal adenocarcinoma and gastric adenocarcinoma cascades and in a range of cell-lines using a custom PyroMark CpG assay. Methylation levels were significantly higher in esophageal tissue with histologically confirmed glandular mucosa as compared to tissue from normal esophagi or gastro-esophageal reflux disease. Higher levels of *OPCML* methylation were absent in the adjacent normal esophageal tissue of patients with glandular mucosa. Higher levels of methylation were confirmed in cell-lines derived from patients with adenocarcinoma, but also detected in two cell-lines with signs of dysplasia. We validated our assay by showing no differences in methylation levels in DNA extracted from blood of patients within the gastric adenocarcinoma cascade. *OPCML* hypermethylation is present in a subset of patients with metaplastic changes in their esophagus.

## Introduction

*OPCML* (Opioid Binding Protein/Cell Adhesion Molecule Like) belongs to the IgLON glycosylphosphatidylinositol (GPI)-anchored cell adhesion molecule family. IgLONs are highly conserved proteins that can anchor to the cell membrane surface by their hydrophobic tails [1, 2]. The expressed OPCML protein follows these patterns where it is localized in the plasma membrane and is highly conserved [3]. Although levels of expression vary, studies have demonstrated OPCML expression in many tissue types, including brain, ovary, heart, placenta, testes, kidney, liver, spleen, pancreas, colon, cervix, prostate, trachea and stomach [1, 2, 4–6], with weak expression in the lung, breast and bone marrow [1].

The CpG island of *OPCML* has been shown to be methylated at relatively low levels in healthy tissue [7]. In contrast, *OPCML* hypermethylation was first associated with epithelial ovarian cancer [7]. This inactivation was later associated with hepatocellular carcinoma [8], lung adenocarcinoma [9] and gastric [10] and brain cancers [2], followed by a variety of other cancers from both primary and metastatic tumors as well as tumor cell lines, including those derived from esophageal adenocarcinoma (EAC) [1]. The hypermethylation of *OPCML* and its consequential transcriptional silencing in a wide variety of cancers is indicative of its role as a tumor suppressor gene (TSG) as well as its role as a potential biomarker for cancer [1, 11]. Further, induced expression in vitro has been found to inhibit the growth of cancer cell lines. Transfection of gastric cancer cell-lines SGC-7901 and BGC-823 with *OPCML*-pcDNA3.1 plasmid resulted in accumulation of cells in the G0/G1 phase and induction of apoptosis [5], suggesting it may also be a therapeutic target.

It has been proposed that OPCML acts as a TSG by interacting with other IgLONs, and loss of this function results in reduced cell adhesion and impairment of signalling between IgLONs [1]. However, McKie et al have shown that OPCML binds the extracellular domains of receptor tyrosine kinases (RTKs), altering their levels, trafficking and downstream signalling, and thus, leading to the inhibition of cellular growth [12].

Here, we examined the methylation levels of *OPCML* locally and systemically in the inflammatory cascades leading to upper gastrointestinal adenocarcinomas.

## Materials and methods

### Recruitment of patients within the esophageal adenocarcinoma cascade

One hundred fifty two predominantly Caucasian subjects who underwent upper gastrointestinal endoscopy at the Prince of Wales Hospital (Sydney) for examination of their gastrointestinal symptoms were recruited prospectively. Subjects who had been prescribed antibiotics or non-steroidal anti-inflammatory drugs in the two-month period prior to recruitment were excluded. A set of two samples were collected at endoscopy, one sample for assessment of *OPCML* methylation locally (esophageal tissue) and another from the same location for histological analysis to be conducted by the pathology services at Prince of Wales Hospital. Histological analysis grouped patients into four groups (normal esophagus, *n* = 89; GERD, *n* = 42; GM, *n* = 20; EAC, *n* = 1). In a subset of patients (GM, *n* = 11), additional samples histologically classified as normal from regions adjacent to the GM region were collected. Further, three samples (normal, GM, and EAC regions) were collected from one patient diagnosed with EAC. All samples were snap-frozen in cryotubes following collection and stored at − 80 °C until processing. Ethics approval was obtained from the South Eastern Sydney Local Health District Human Research Ethics Committee (HREC 13/375 and HREC 16/020). All subjects recruited to the study signed a written informed consent, and all experiments were performed in accordance with relevant guidelines and regulations.

### Recruitment of patients within Correa’s (gastric adenocarcinoma) cascade

The gastric adenocarcinoma (GAC) cascade study population comprises Mestizo individuals (54 non-cardia GAC cases and 55 age-matched functional dyspepsia controls) from Pereira, a city located in the Colombian Andes mountain range, who underwent upper gastrointestinal endoscopy at Comfamiliar Risaralda Hospital. Patients known to be infected with the Human Immunodeficiency Virus, who have any comorbidity associated with immunosuppression or who had been prescribed non-steroidal anti-inflammatory drugs, anti-microbial agents or acid suppressants in the three-month period prior to recruitment were excluded. As Colombian individuals present with GAC early in life, subjects > 30 years were recruited, the critical age from which the incidence increases steadily thereafter in this population. In addition, 91 subjects with gastric precancerous lesions composed of 40 subjects with atrophic gastritis, 40 subjects with intestinal metaplasia and 11 subjects with dysplasia, based on histological assessment by experienced pathologists, have been included. In addition to gastric biopsies for histological assessment, a blood sample for assessment of *OPCML* methylation systemically was collected at endoscopy. Blood samples (5 ml) were collected intravenously in BD Vacutainer® SST™ Blood Collection Tubes and immediately stored at − 20 °C until they were shipped frozen on dry ice to Australia. Samples were then stored at − 20 °C until processing. Ethics approvals have been gained from Comfamilar Risaralda Hospital (Acta 5140) and the University of New South Wales (UNSW) (HREC 16010) human research ethics committees.

### Growth and maintenance of human cell lines

HET-1A cells were grown in 10 ml cell culture media comprising LHC-9 media (Catalog no. 12680013, ThermoFisher Scientific; Scoresby, Vic, Australia) supplemented with 10% heat-inactivated fetal bovine serum (hi-FBS) and 100 μg/ml penicillin/streptomycin, in 25 cm^2^ tissue culture flasks at 37 °C with 5% CO_2_. CP-A, CP-B, CP-C, and CP-D were grown in Keratinocyte-SFM media (Catalog no. 17005042, ThermoFisher Scientific) supplemented with prequalified human recombinant Epidermal Growth Factor 1–53 (5 μg/l), Bovine Pituitary Extract (50 mg/l), 10% hi-FBS and 100 μg/ml penicillin/streptomycin. OE33, AGS, and LS 174 T cells were grown in Roswell Park Memorial Institute (RPMI) media supplemented with 10% hi-FBS and 100 μg/ml penicillin/streptomycin. Caco-2 cells were grown in Minimum Essential Medium supplemented with 10% hi-FBS, 1 mM sodium pyruvate, 0.1 mM non-essential amino acids, 2.25 mg/1 sodium bicarbonate and 100 μg/ml penicillin/streptomycin.

### DNA extraction

Our recruitment of the two study cohorts resulted in two types of samples: 1) mucosal samples from the esophagus of subjects recruited to the EAC cascade cohort; 2) blood samples collected intravenously from subjects recruited to the GAC cascade cohort. DNA was extracted from esophageal samples and human cell-lines using Gentra Puregene Tissue kit (Qiagen; Chadstone Centre, Vic, Australia) according to the manufacturer’s instructions. DNA was extracted from blood samples using the QIAamp DNA Blood Mini Kit (Qiagen) according to the manufacturer’s instructions.

### Custom OPCML methylation assay

The custom *OPCML* methylation assay was performed using services and facilities available at the Australian Genome Research Facility Ltd. All pyrosequencing assays were designed using the algorithms built into the PyroMark Assay Design Software (Version 2.0.1, Qiagen). Briefly, approximately 200 bp of reference sequence surrounding the target CpG sites (*OPCML* exon 1) were inputted into the software. CpG sites were selected as target sites for analysis and primers designed to target these sites were chosen from a list generated by the software on the basis of the algorithms’ predicted assay quality. The forward PCR primer for the *OPCML* assay was TTGGGATGAAGAGTAGGGTAGT, the reverse PCR primer was 5′Biotin-CTCCCTCCCTTTACAAACATT, and the sequencing primer was TGAAGAGTAGGGTAGTT.

DNA samples were converted using the Epitect Bisulphite Conversion Kit (Qiagen). 500 ng of genomic DNA were converted overnight in a total volume of 140 μl using the standard protocol from the kit. Converted DNA was isolated on provided columns and stored at − 20 °C. The assay regions containing the CpG target sites were PCR amplified using a biotin labelled, HPLC purified primer and standard sequencing grade primer. All PCR amplifications were performed with the PyroMark PCR Kit (Qiagen). Amplification reactions consisted of 12.5 μl PyroMark Mastermix, 2.5 μl Coral Load, 1 μl of each of 5 μM forward and reverse primers, 2 μl of bisulphite converted template DNA, and 6 μl of water. Thermocycling conditions consisted of 15 min at 95 °C followed by 45 cycles of 30 s at 95 °C, 30 s at 56 °C and 30 s at 72 °C and a final extension step of 10 min at 72 °C. All amplifications were visualised on 2% agarose gels to confirm quality and estimate concentration.

The PCR product was bound to Streptavidin Sepharose High Performance beads (GE Healthcare Life Sciences; Parramatta, NSW, Australia), the beads containing the immobilized PCR product were denatured and washed using proprietary solutions (Qiagen) on the Pyrosequencing Vacuum Prep Tool (Qiagen) to isolate a single stranded template. The beads were then transferred to an optically clear, 24-well sequencing plate in 0.3 μM of pyrosequencing primer. Annealing to the single-stranded template was done by heating the plate to 80 °C followed by cooling to room temperature. Pyrosequencing was performed on a PyroMark 24 Pyrosequencing System (Qiagen) as per the manufacturer’s instructions. Data was analyzed on the PyroMark Q24 software to give the methylation values (%) for each CpG site in the sample.

### Statistics

Comparisons of *OPCML* methylation levels across patient groups were performed using one-way ANOVA with post hoc Tukey’s test and false discovery rate correction where applicable.

## Results

### OPCML methylation in esophageal samples of patients within the esophageal adenocarcinoma cascade

**Fig. 1:**
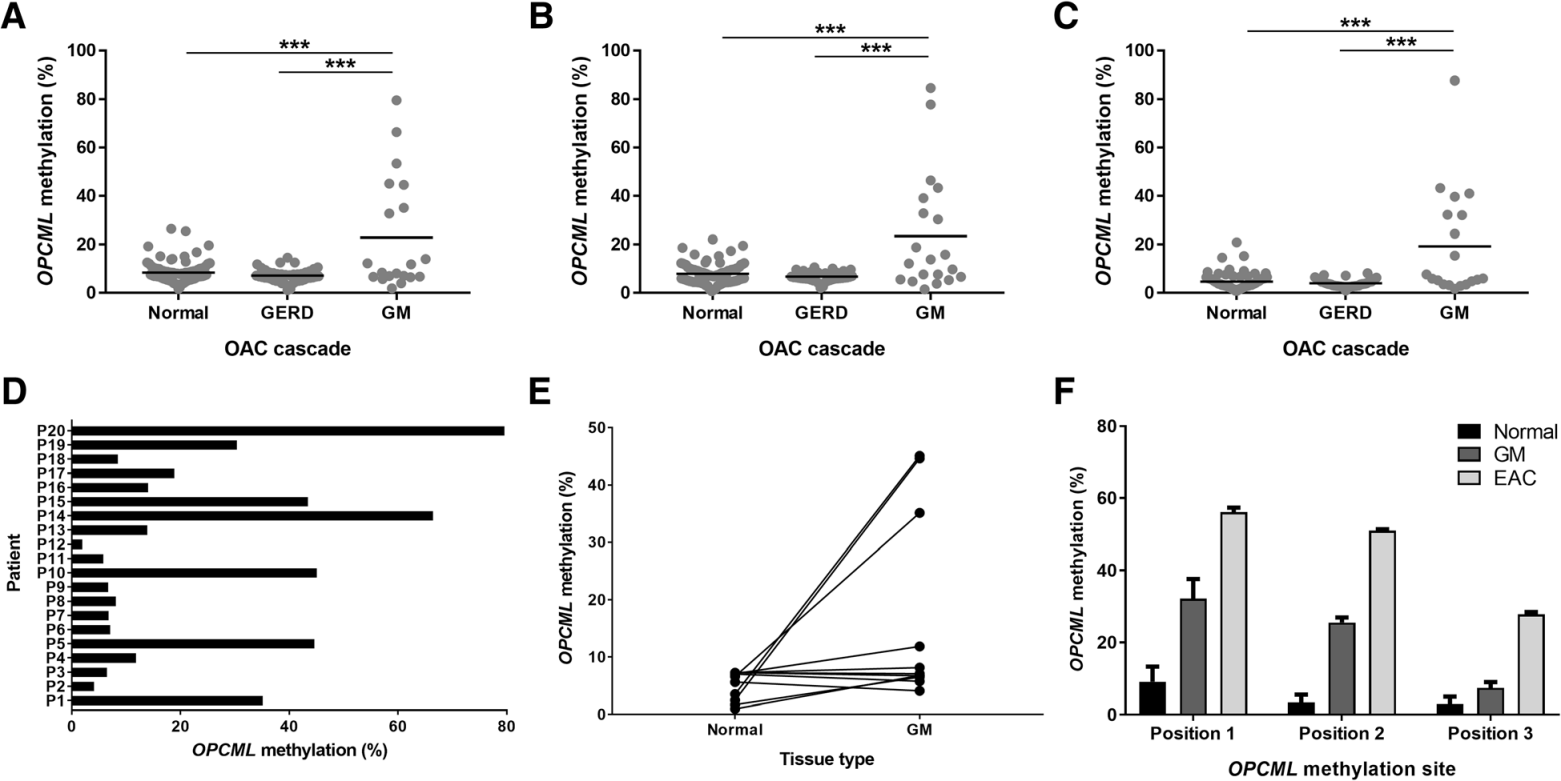
*OPCML* methylation in normal esophageal samples or from patients within the EAC cascade. Position 1 (**a**), position 2 (**b**), and position 3 (**c**) within the first exon of *OPCML*. **d** *OPCML* methylation at position 1 across the 20 patients with GM. Site of GM was analyzed. Position 1 was shown as representative results across the three positions. **e** *OPCML* methylation at position 1 across the 11 patients with GM. Both normal and GM tissue were analyzed from the same patient. Patients corresponded to P1–11 in Panel D. Patterns were consistent for positions 2 and 3. **f** *OPCML* methylation at positions 1, 2 and 3 for the normal, GM and EAC tissues within the same EAC patient (*n* = 3 replicates across all measurements). Position 1: hg19_chr11:133402185; CpG site: cg19151121. *: *P* < 0.05; **: *P* < 0.01; ***: *P* < 0.001. P: Patient; GERD: gastro-esophageal reflux disease; GM: detectable glandular mucosa; EAC: esophageal adenocarcinoma

Methylation levels of three CpG sites within exon 1 of *OPCML* (position 1: hg19_chr11:133402185; CpG site: cg19151121) were measured in esophageal samples from patients within the EAC cascade. We observed significantly higher levels of methylation across the three positions in patients that had histologically detectable glandular mucosa (GM) as compared to patients with a normal esophagus and those with gastro-esophageal reflux disease (GERD) (Fig. 1a-c). However, on further analysis, it was clear that there was no consistent methylation pattern across the GM group (Fig. 1d), and the significant differences with respect to normal and GERD patients were driven by intermediate to high methylation patterns in a specific subgroup of GM patients.

Importantly, of the 20 patients with GM, normal esophageal tissue samples (confirmed histologically) from 11 of the patients were collected from regions adjacent to the GM region. No hypermethylation was observed in the normal tissue from these patients (Fig. 1e).

As part of the prospective enrolment of subjects, one EAC patient was recruited. During endoscopy, the patient was visually suspected to have an EAC, and thus, three samples were collected from regions that were later histological confirmed as normal, GM and EAC. OPCML methylation was confirmed to show a step-wise increase across the three sites and three positions (Fig. 1f).

### OPCML methylation in human cell-lines

**Fig. 2:**
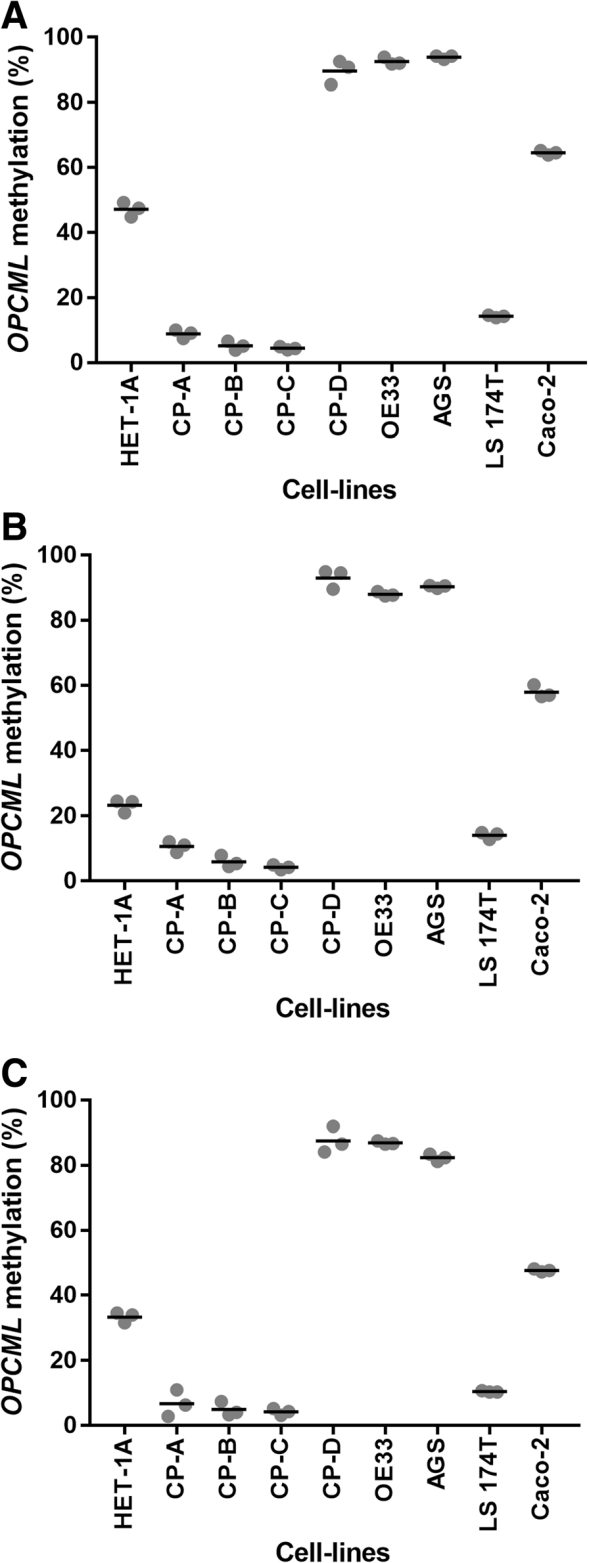
*OPCML* methylation in human cell-lines. Position 1 (**a**), position 2 (**b**), and position 3 (**c**) within the first exon of *OPCML*. Three biological replicates were performed. Position 1: hg19_chr11:133402185; CpG site: cg19151121

Given the signature observed in the esophageal samples from patients within the EAC cascade, we then measured methylation levels of the same three sites within *OPCML* in nine esophageal, gastric and intestinal cell-lines. Methylation patterns were consistent across all three positions (Fig. 2a-c). Cell-lines derived from upper gastrointestinal adenocarcinomas (OE33 and AGS) were methylated higher as compared to those derived from lower gastrointestinal adenocarcinomas (LS 174 T and Caco-2; Fig. 2). Despite the fact that EAC cascade cell-lines CP-B, CP-C and CP-D are derived from areas of high grade dysplasia while CP-A is derived from an area of nondysplastic metaplasia [13, 14], we observed low levels of methylation for CP-A, CP-B and CP-C when compared to CP-D (Fig. 2). In fact, CP-D showed similar methylation levels to the EAC cell-line OE33. Surprisingly, we observed intermediate levels of *OPCML* methylation in immortalized primary normal esophageal cells HET-1A (Fig. 2).

### OPCML methylation in blood of patients within the gastric adenocarcinoma cascade

**Fig. 3:**
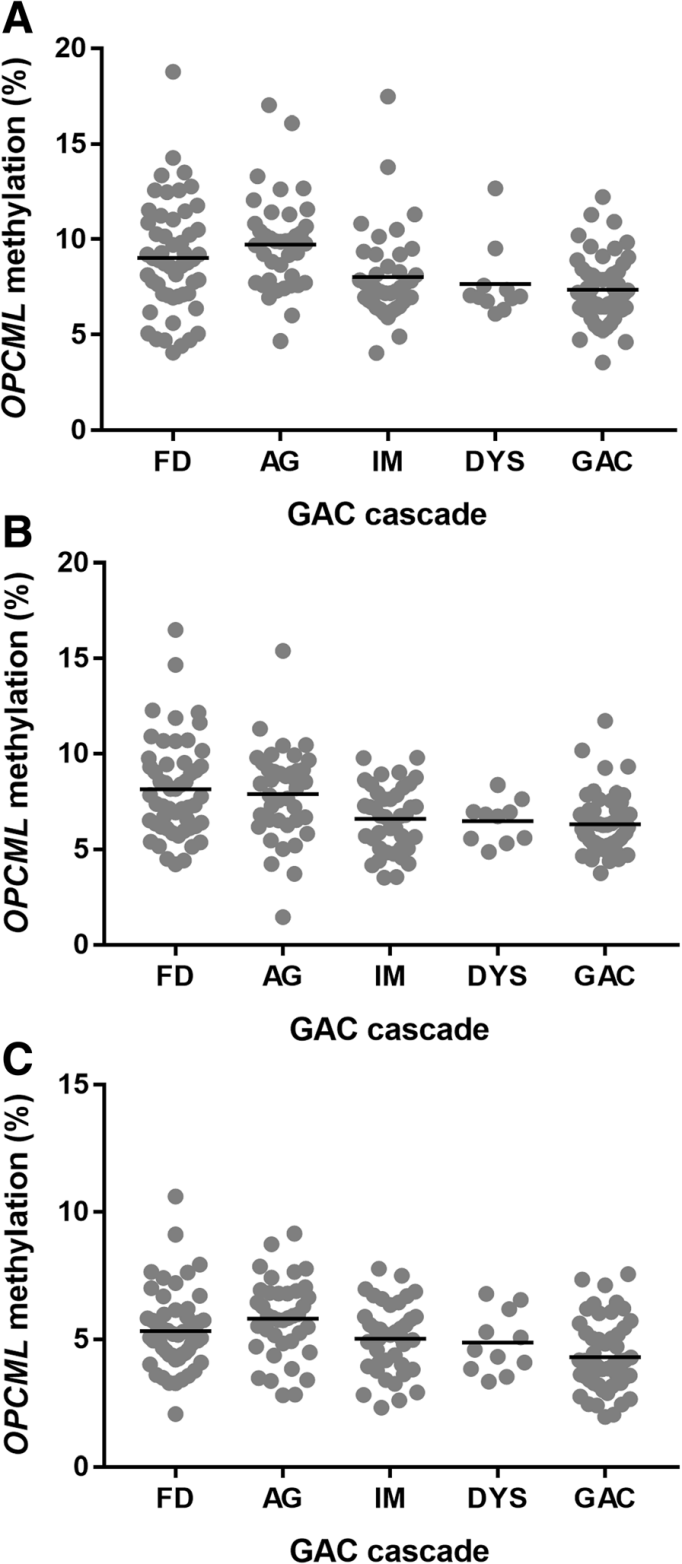
*OPCML* methylation in blood of patients within the GAC cascade. Position 1 (**a**), position 2 (**b**), and position 3 (**c**) within the first exon of *OPCML*. Three technical replicates of each patient sample were performed. Data presented are of one technical replicate and are representative of all repeats. FD: functional dyspepsia; AG: atrophic gastritis; IM: intestinal metaplasia; DYS: dysplasia; GAC: gastric adenocarcinoma

Given that peripheral blood mononuclear cells (PBMCs) do not express *OPCML* [1] and *OPCML* is hypermethylated in GAC tissue [10], we then recruited a cohort of patients across the GAC cascade (*n* = 200) and collected blood intravenously. This was performed to validate our PyroMark assay and local tissue results in a large cohort of patients within an inflammatory adenocarcinoma cascade employing samples (i.e. blood) that should not show elevated methylation levels of *OPCML*. *OPCML* methylation was measured in DNA extracted from blood samples (Fig. 3). *OPCML* methylation of exon 1 fluctuated around levels detected in normal tissues (mean (%) ± SD; position 1: 8.4 ± 2.5; position 2: 7.2 ± 2.2; position 3: 5.1 ± 1.5). No differences in *OPCML* methylation in blood derived from patients across the GAC cascade were observed (Fig. 3a-c).

## Discussion

*OPCML* promoter epigenetic silencing in the form of hypermethylation has been proposed as a potential biomarker of a range of cancers [1, 7–11]. Here, we show that intermediate levels of *OPCML* exon 1 methylation in the local tissue occurs in a subset of individuals with metaplasia.

Patients within the EAC cascade were recruited and *OPCML* methylation levels were examined in esophageal tissue samples. Significant differences in *OPCML* methylation patterns were observed between patients with GM as compared to patients with normal esophagi and GERD. These differences were found to be driven by intermediate and high methylation patterns within individual patients rather than a consistent methylation pattern across the entire GM subgroup. Further, hypermethylation was not detected in normal esophageal tissue from patients with GM. We suggest that *OPCML* hypermethylation in these patients could suggest early signs of dysplastic events not detected histologically. While arising from only one patient, and thus, should be assessed with caution, the importance of *OPCML* hypermethylation is supported by our comparisons across the EAC histological cascade within one EAC patient.

In esophageal epithelial cell-lines across the EAC cascade, high levels of methylation were observed in one of the three cell-lines derived from dysplastic Barrett’s esophagus (BE) patients as well as in the cell-line derived from an EAC patient. Methylation levels similar to those in normal tissue were observed in the cell-line derived from non-dysplastic BE. Intriguingly, HET-1A, an immortalized esophageal epithelial cell-line derived from normal squamous tissue showed intermediate levels of *OPCML* methylation. This is highly relevant considering Underwood et al. have shown HET-1A cells appear dysplastic and fail to display evidence of squamous differentiation [15]. This data would further suggest that *OPCML* hypermethylation is associated with specific dysplastic events in esophageal tissue, and HET-1A will be a useful cell-line to delineate the events that lead to *OPCML* hypermethylation.

The assay in this study was designed to target three sites within the first exon of OPCML for two reasons. First, we set out to design a rapid assay that can be translated into clinical applications in the future. Second, hypermethylation of the first exon of genes has been reported to be more tightly linked to transcriptional silencing than methylation in the promoter region [16]. To validate our assay and local tissue results, a large cohort of patients within the GAC cascade was recruited and blood collected. No hypermethylation of *OPCML* was observed in any of the disease groups, and this is consistent with the lack of expression of OPCML in PBMCs [1]. The assay also showed high consistency across the 200 subjects within this cohort. Given that hypermethylation of the *OPCML* promoter has been linked to downregulation of expression [5], further validation of the effects of hypermethylation of *OPCML* exon 1 should come in the form of OPCML expression profiling in local tissues. Moreover, there is a need to determine if OPCML has similar effects on receptor tyrosine kinases in the EAC cascade as has been observed in ovarian cancer [12, 17].

In conclusion, *OPCML* hypermethylation appears to be a signature that should be investigated further in carcinogenesis. Additional studies assessing larger cohorts are required to confirm the importance of *OPCML* methylation prior to tumor development.

## Abbreviations

BE: Barrett’s esophagus
CpG: Cytosine-phosphate-guanine
EAC: Esophageal adenocarcinoma
GAC: Gastric adenocarcinoma
GERD: Gastro-esophageal reflux disease
GM: Glandular mucosa
GPI: Glycosylphosphatidylinositol
HREC: Human Research Ethics Committee
OPCML: Opioid Binding Protein/Cell Adhesion Molecule Like
RTK: Receptor tyrosine kinase
TSG: Tumor suppressor gene
